# Epstein-Barr Virus in Burkitt Lymphoma in Africa Reveals a Limited Set of Whole Genome and *LMP-1* Sequence Patterns: Analysis of Archival Datasets and Field Samples From Uganda, Tanzania, and Kenya

**DOI:** 10.3389/fonc.2022.812224

**Published:** 2022-03-07

**Authors:** Hsiao-Mei Liao, Hebing Liu, Pei-Ju Chin, Bingjie Li, Guo-Chiuan Hung, Shien Tsai, Isaac Otim, Ismail D. Legason, Martin D. Ogwang, Steven J. Reynolds, Patrick Kerchan, Constance N. Tenge, Pamela A. Were, Robert T. Kuremu, Walter N. Wekesa, Nestory Masalu, Esther Kawira, Leona W. Ayers, Ruth M. Pfeiffer, Kishor Bhatia, James J. Goedert, Shyh-Ching Lo, Sam M. Mbulaiteye

**Affiliations:** ^1^ Office of Tissues and Advanced Therapies, Center for Biologics Evaluation and Research, Food and Drug Administration, Silver Spring, MD, United States; ^2^ EMBLEM Study, St. Mary’s Hospital, Lacor, Gulu & African Field Epidemiology Network, Kampala, Uganda; ^3^ EMBLEM Study, African Field Epidemiology Network, Kampala, Uganda; ^4^ EMBLEM Study, Kuluva Hospital, Arua & African Field Epidemiology Network, Kampala, Uganda; ^5^ Division of Intramural Research, National Institute of Allergy and Infectious Diseases, National Institutes of Health, Bethesda, MD, United States; ^6^ EMBLEM Study, Moi University College of Health Sciences, Eldoret, Kenya & Academic Model Providing Access To Healthcare (AMPATH), Eldoret, Kenya; ^7^ EMBLEM Study, Bugando Medical Center, Mwanza, Tanzania; ^8^ EMBLEM Study, Shirati Health and Educational Foundation, Shirati, Tanzania; ^9^ Department of Pathology, The Ohio State University, Columbus, OH, United States; ^10^ Division of Cancer Epidemiology and Genetics, National Cancer Institute, National Institutes of Health, Bethesda, MD, United States

**Keywords:** epidemiology, LMP-1 patterns, EBV variants, Burkitt lymphoma, childhood cancer, Epstein-Barr virus, East Africa

## Abstract

Epstein-Barr virus (EBV) is associated with endemic Burkitt lymphoma (eBL), but the contribution of EBV variants is ill-defined. Studies of EBV whole genome sequences (WGS) have identified phylogroups that appear to be distinct for Asian versus non-Asian EBV, but samples from BL or Africa, where EBV was first discovered, are under-represented. We conducted a phylogenetic analysis of EBV WGS and *LMP-1* sequences obtained primarily from BL patients in Africa and representative non-African EBV from other conditions or regions using data from GenBank, Sequence Read Archive, or Genomic Data Commons for the Burkitt Lymphoma Genome Sequencing Project (BLGSP) to generate data to support the use of a simpler biomarker of geographic or phenotypic associations. We also investigated *LMP-1* patterns in 414 eBL cases and 414 geographically matched controls in the Epidemiology of Burkitt Lymphoma in East African children and minors (EMBLEM) study using *LMP-1* PCR and Sanger sequencing. Phylogenetic analysis revealed distinct genetic patterns of African versus Asian EBV sequences. We identified 281 single nucleotide variations (SNVs) in *LMP-1* promoter and coding region, which formed 12 unique patterns (A to L). Nine patterns (A, AB, C, D, F, I, J, K and L) predominated in African EBV, of which four were found in 92% of BL samples (A, AB, D, and H). Predominant patterns were B and G in Asia and H in Europe. EBV positivity in peripheral blood was detected in 95.6% of EMBLEM eBL cases versus 79.2% of the healthy controls (odds ratio [OR] =3.83; 95% confidence interval 2.06-7.14). *LMP-1* was successfully sequenced in 66.7% of the EBV DNA positive cases but in 29.6% of the controls (ORs ranging 5-11 for different patterns). Four *LMP-1* patterns (A, AB, D, and K) were detected in 63.1% of the cases versus 27.1% controls (ORs ranges: 5.58-11.4). Dual strain EBV infections were identified in WGS and PCR-Sanger data. In conclusion, EBV from Africa is phylogenetically separate from EBV in Asia. Genetic diversity in *LMP-1* formed 12 patterns, which showed promising geographic and phenotypic associations. Presence of multiple strain infection should be considered in efforts to refine or improve EBV markers of ancestry or phenotype.

**Lay Summary:**

Epstein-Barr virus (EBV) infection, a ubiquitous infection, contributes to the etiology of both Burkitt Lymphoma (BL) and nasopharyngeal carcinoma, yet their global distributions vary geographically with no overlap. Genomic variation in EBV is suspected to play a role in the geographical patterns of these EBV-associated cancers, but relatively few EBV samples from BL have been comprehensively studied. We sought to compare phylogenetic patterns of EBV genomes obtained from BL samples in Africa and from tumor and non-tumor samples from elsewhere. We concluded that EBV obtained from BL in Africa is genetically separate from EBV in Asia. Through comprehensive analysis of nucleotide variations in EBV’s *LMP-1* gene, we describe 12 *LMP-1* patterns, two of which (B and G) were found mostly in Asia. Four *LMP-1* patterns (A, AB, D, and F) accounted for 92% of EBVs sequenced from BL in Africa. Our results identified extensive diversity of EBV, but BL in Africa was associated with a limited number of variants identified, which were different from those identified in Asia. Further research is needed to optimize the use of PCR and sequencing to study *LMP-1* diversity for classification of EBV variants and for use in epidemiologic studies to characterize geographic and/or phenotypic associations of EBV variants with EBV-associated malignancies, including eBL.

## Introduction

Epstein-Barr virus (EBV) was discovered in cultures of tumor cells of a child with jaw sarcomas, subsequently named endemic Burkitt lymphoma (eBL), in 1964 (1). EBV’s association with eBL was confirmed in a prospective study conducted in Uganda in 1978 (2), and in 1997 it was declared a Class 1 carcinogen for BL (3), and for other cancers where it is consistently detected. These cancers include nasopharyngeal carcinoma (NPC) (4), NK/T-cell lymphomas (5), Hodgkin lymphoma (HL), post-transplant proliferative disease (PTLD), and gastric cancer (GC) (6). Recent studies have confirmed that EBV status represents distinct molecular landscapes of the associated cancers, including BL (7), GC (8), and NPC (9). In 2017, EBV-associated cancers accounted for 256,000 cancers globally and 164,000 cancer deaths (18% and 17%, respectively) (6).

EBV infects >95% of adults globally (10, 11), but BL and NPC exhibit distinct geographic distributions and age-specific patterns that are unexplained by simple EBV epidemiology. BL is the commonest childhood cancer in equatorial Africa and Papua New Guinea where 5-10 per 100,000 children below 15 years are affected (12) and is rare elsewhere. NPC occurs with high incidence in Eastern and South-Eastern-Asia and in some areas of the Middle East and North Africa (4). These distinct geographical patterns of BL and NPC could theoretically be attributed to genomic variations of EBV circulating in the different world regions. The discovery of variations in *EBNA-2* and *EBNA-3* genes enabled the classification of EBV into types 1 and 2 (13, 14), with apparently different distributions in EBV isolates worldwide. However, a literature review of studies conducted up to 2009 showed that none of the genetic variations in EBV studied up to that point were either associated with EBV-associated malignancies or could explain the geographic patterns of the malignancies (4). Some of the possible limitations of the studies reviewed included focusing on variation in single EBV genes, such as *LMP-1* (15, 16), *EBNA-1* (17), or *BZLF-1* (18), because they were linked to suggestive biological properties of transformation (19), but no convincing epidemiological associations with disease patterns emerged (4).

The successful whole genome sequencing (WGS) of EBV samples (13, 20, 21) and increasing access to high-throughput sequencing (HTS) data of EBV from tumor and non-tumor samples present new opportunities to investigate genomic variations of EBV that may be associated with EBV-associated cancers. HTS studies have been utilized to discover genomic variations in EBV associated with NPC (22, 23) and to investigate EBV genomic variations in samples from regions that were previously underrepresented, such as South America (24), and genomic variations in EBV from Africa or from BL (25).

We previously reported (25) 51 novel single nucleotide variants (SNVs) in the sequence spanning a 2.1 kb region of the *LMP-1* promoter and coding region Exon 1-3 in 13 of 14 of primary BL biopsies from Ghana, Brazil, and Argentina that were investigated using HTS. The SNVs formed four unique *LMP-1* patterns when aligned for the 112 EBV genomic samples available in GenBank, comprising 23, 29, and 3 shared SNVs in the promoter, *LMP-2B* Exon 1, and *LMP-1* Exon 1 regions, respectively. The nucleotide variation patterns in *LMP-1* were labeled A, B, and C, and the samples with the wild type (WT) reference sequence were labeled pattern D (25). EBV pattern A was observed in 48% of the 27 EBV samples from BL samples (primary biopsies or BL-derived cell lines) in GenBank but only in 8% of 85 non-BL samples analyzed (25). Pattern A variations were validated in the primary BL biopsies using Sanger sequencing of PCR products using 3 primer sets (Lei-1, 2, and 3) designed to capture the whole 2.1 kb hypervariable region in *LMP-1* promoter and coding regions (25). Pattern A was the most frequently detected pattern among 50 additional BL tumors from Ghana, Argentina, and Brazil subsequently tested (25, 26), highlighting this pattern as being frequent in BL or samples from Africa.

The discovery of novel *LMP-1* patterns (25) builds on findings from previous studies of genetic diversity of *LMP-1* (27–32), which is widely accepted as an EBV oncogene (15, 16). Some of these studies have suggested ways to classify and study EBV genetic diversity, such as the 30 bp deletion in the C-terminus (28), the loss of restriction site XhoI in the N-terminus of *LMP-1* (29), and the classification proposed by Edwards et al. based on nucleotide variants resulting in signature amino acid changes in the C-terminus of *LMP-1* relative to the WT (B95-8) (27). The Edward's classification includes seven variants named according to the geographic region from which the initial EBV isolate was originally derived, such as Alaskan, China 1, China 2, China 3, Mediterranean + (Med +), Med -, and North Carolina. These classification systems have been used to study the biology of EBV, but as reviewed in Chang et al. (4), their utility as biomarkers of geographic or of cancer phenotypic associations has been less clear. Because the geographic patterns of BL or NPC in endemic versus non-endemic areas vary 30-90-fold (33), a useful marker for study of the geographic or phenotypic associations with EBV should be rare in geographical areas where the associated cancers are rare and common in geographical areas where the associated cancers also are common.

To guide our further epidemiological research using the novel *LMP-1* patterns reported in Lei et al. (25) versus other established classifications of *LMP-1* diversity (27–32), we performed comparative analysis of EBV genetic variation using the Lei patterns versus seven other systems for 114 EBV sequences that were used in the discovery study by Lei et al. (25) and Liao et al. (26). Our comparative analysis confirmed that the SNVs used to define patterns in Lei et al. did not overlap with nucleotide positions in the regions used to classify EBV in the seven other systems. Most of those systems utilized amino acid changes coded by nucleotides in the 3^rd^ Exon of *LMP-1* (26). The system proposed by Edwards *et al.* yielded a reasonable representation of phylogenetic clusters (23, 27), but it did not allow a clear geographic separation of samples of African from those of Asian origin, whereas the patterns proposed by Lei et al. do (25). Similarly, the 30 bp pair loss in the C-terminus (28), although easy to classify, did not discriminate either phenotypic or geographic associations (26).

Here, we expand our results of studying *LMP-1* patterns through phylogenetic analysis of the largest set of EBV whole genome sequences (WGS) focusing primarily on samples from BL or from Africa. We also report a detailed curation of pattern-forming SNVs in *LMP-1* and analyze them in the context of EBV samples from all other conditions and from elsewhere. To get preliminary data about *LMP-1* patterns in eBL cases and age- and -geographically matched healthy controls for comparisons, we also performed targeted PCR and Sanger sequencing of *LMP-1* in peripheral blood samples of 414 childhood eBL cases and 414 geographically matched non-BL controls obtained in the Epidemiology of Burkitt Lymphoma in East African children and minors (EMBLEM) study (34).

## Methods

### EBV Samples Compiled to Study Genomic Variations

**Figure 1:**
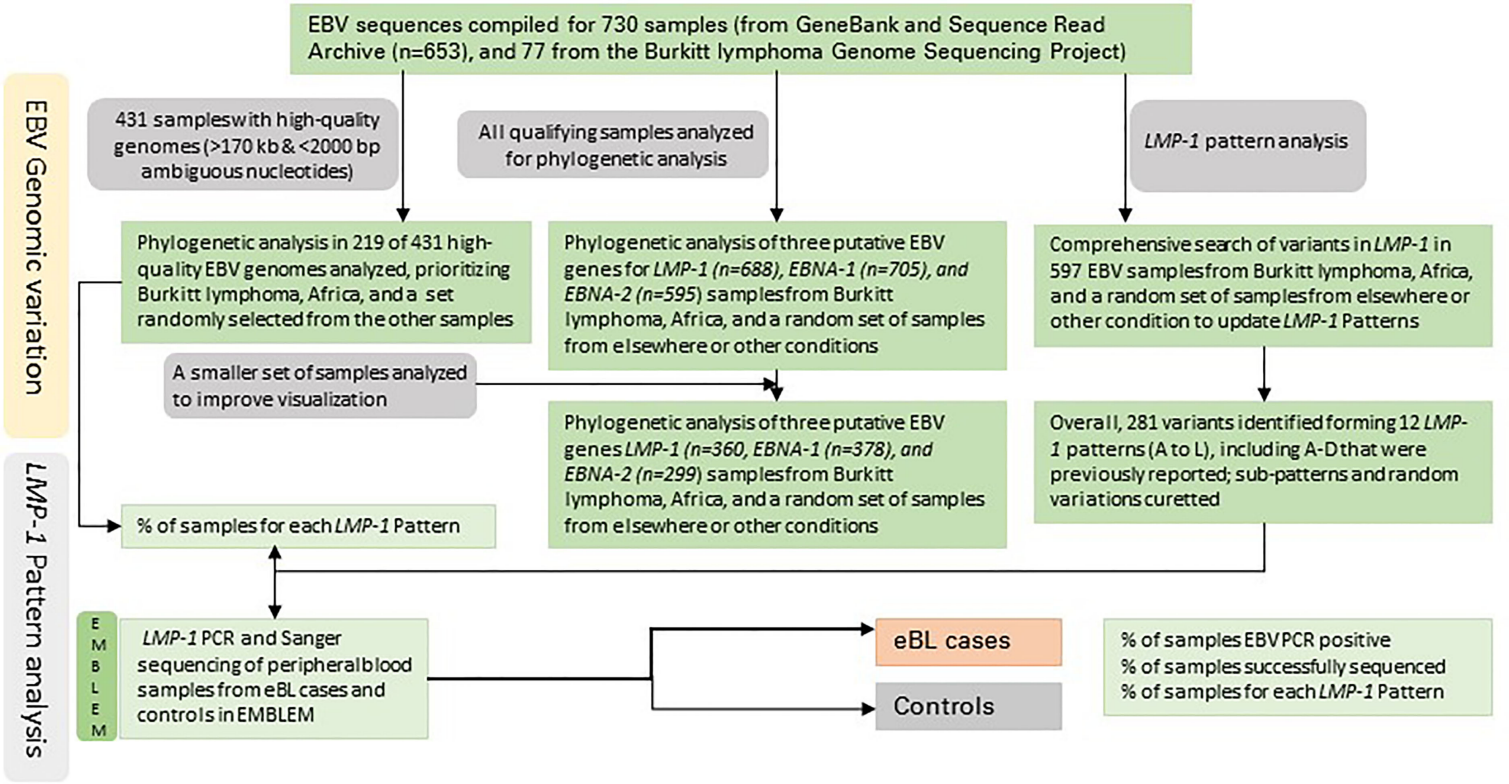
The workflow of the EBV genomic variation identification and pattern analysis.

**Figure 2:**
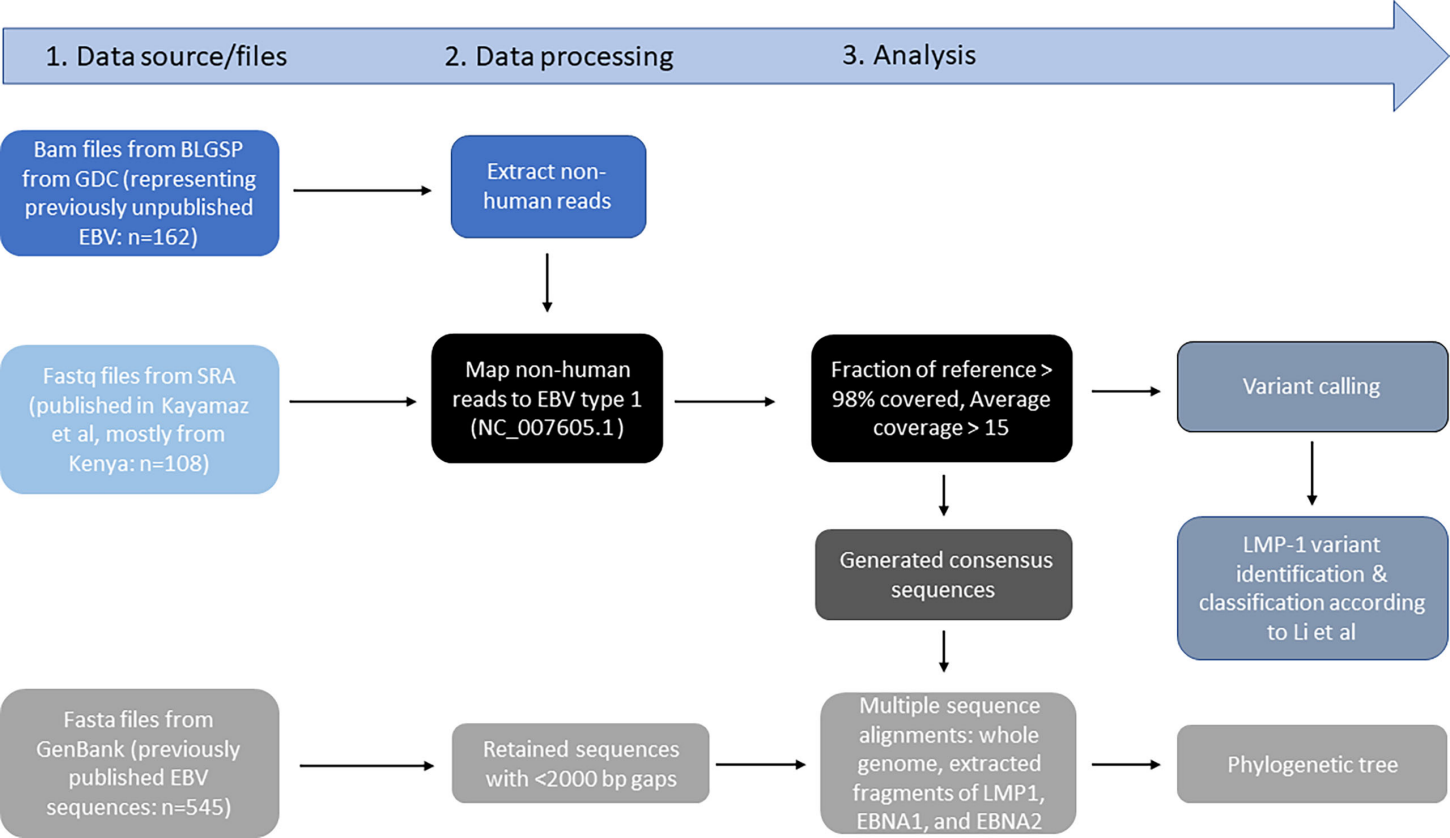
Data source files, data processing, and analysis workflow.

**Figures 1**, **2** show the samples selection and prioritization flow and the data sources, processing, and analysis flow charts. We accessed 730 EBV genomic sequences obtained from BL, HL, NK/T lymphoma, PTLD, NPC, GC, infectious mononucleosis (IM), lymphoblastoid cell lines (LCL), and healthy donors and diverse geographic regions. The previously published EBV genomic sequences (*n*=545) were accessed as fasta files from GenBank or fastq files from the Sequence Read Archive (SRA) (35) (*n*=108; **Supplementary Table S1**) The sequence metadata, including accession numbers, sample type, geographical area, country, and EBV type were downloaded (35). The new EBV sequences from the Burkitt Lymphoma Genome Sequencing Project (BLGSP) were accessed as Bam files from Genomic Data Commons using the GDC Data Transfer (GDC, https://portal.gdc.cancer.gov/; Project ID: CGCI-BLGSP, dbGap study accession: phs000527.v13.p4; details in **Supplementary Table S2**). A total of 162 files were accessed, of which 77 had high-quality EBV content and were analyzed (7). The fasta files from GenBank were consensus EBV sequences and were filtered to exclude low-quality sequences, defined as having an in-house calculated N number > 2000, assuming a sequencing error rate of 1% and genome length >170 Kb. The genomic sequences from the BLGSP were flagged by default as mapped to the human reference genome (GRCh38) or unmapped. The reads mapped to the human genome were removed by Bowtie2. The unmapped reads, which are considered non-human, were extracted from the BAM files using command view -@ 20 -f 12 -F 256 in Samtools on NIH HPC Biowulf cluster and imported into CLC Genomics Workbench version 20.0.4 (Qiagen Bioinformatics, USA) as fastq files and mapped to the EBV wildtype (WT) B95-8 reference genome (NC_007605.1) following the same approach described in Lei et al. (25) using default parameters of Map Reads to Reference tool. To minimize the inclusion of low-quality results, we filtered out sequences with an average read depth <15 and <98% coverage of the EBV reference genome, resulting in 77 high-quality, full-length EBV genomes. These genomes were subjected to variant calling using Fix Ploidy Variant Detection tool of CLC Genomics Workbench. Overall, 431 of the 730 compiled EBV sequences were deemed high-quality for multiple sequence alignment (MSA) for phylogenetic analysis.

We primarily focused on 219 (**Supplementary Table S3**), including all good quality WGS from 130 BL and Africa (74 new EBV genomic sequences from the BLGSP) plus 89 representative sequences sampled by EBV phylogenetic clade among the non-African origin samples. Because the non-African EBV from certain regions, particularly Asia, were many, these sequences were sampled by clade from the aligned sequence in the alignment files in fasta with a cap placed at 35% for sequences belonging to large clades. EBV type was based on metadata, except for those samples where EBV type was recorded as “unknown” for which EBV type was assigned by aligning *EBNA2* sequences. EBV type could not be determined for small number of EBV genomic datasets with poor *EBNA2* sequences, which remained undetermined in our analyses. The 219 complete genomes were aligned by MAFFT v7 (36), installed on the NIH high-performance computing (HPC) Biowulf cluster, and the MSA file (https://github.com/smbulaiteye/EBVBL_Africa_focus.git) was used to construct unrooted phylogenetic trees using the Neighbor-Joining (NJ) and the Jukes-Cantor method to measure the genetic distance of the aligned sequences. Although the NJ method may not be optimal for calculating the phylogenetic distance or accurately characterizing consequences of evolutionary diversification (37), this was not the focus of our paper because EBV evolution has been addressed in several recent excellent reports (24, 38–41). We utilized the NJ method because it has reasonable performance and accuracy for studies of genotype clades and geographic and/or phenotype patterns (42). We conducted a phylogenetic analysis of *LMP-1* sequences to determine to the concordance between WGS and *LMP-1* patterns. Additionally, we conducted a limited parallel phylogenetic analysis of *EBNA-1* and *EBNA-2* as a sensitivity analysis about the specificity of *LMP-1* patterns.

MSAs for *LMP-1, EBNA-1,* and *EBNA-2* (https://github.com/smbulaiteye/EBVBL_Africa_focus.git) were generated using the ClustalW algorithm in BioEdit (v7.0). The internal repetitive region of the *EBNA-1* gene was excluded from the alignment. Phylogenetic analysis used the Neighbor-Joining algorithm and the Jukes-Cantor method. These gene-specific analyses allowed us to include more samples than were possible for WGS samples. These analyses were conducted using the largest number of sequences that qualified (with a calculated N<10 N in the aligned genes) and repeated for a smaller set of samples to allow better visualization of the phylogenetic patterns and to assess whether the patterns observed in the full set of sequences remained apparent in the same in the smaller set, i.e., selecting fewer samples does not obviously bias the patterns. Thus, for the full set, we analyzed 668 *LMP-1* sequences (listed in **Supplementary Table S4** **),** 705 *EBNA-1* sequences (listed **Supplementary Table S5**) and 595 *EBNA-2* sequences (listed in **Supplementary Table S6**). These analyses were repeated for a smaller set of samples **(** **Supplementary Tables S7–S9** for *LMP-1*, *EBNA-1*, and *EBNA-2*, respectively**)**. These subset samples were selected to include all samples with qualifying read depth and coverage (see above) from BL patients or from Africa and a set of non-BL, non-African samples selected by clade as described above.

The 2.1 kb *LMP-1* promoter and coding region (Exon 1-3) was carefully curated for SNVs in 597 samples (185 from BL or from Africa, including 77 new sequences from the BLGSP, 40 controls from Africa, and 412 non-BL samples reported from elsewhere) to identify pattern forming *LMP-1* variants. Synonymous variants in the *LMP-1* coding regions and intronic variants were not used to classify variant patterns (**Supplementary Table S10** shows the genotyping results in representative samples).

### *LMP-1* Patterns in Peripheral Blood of Cases and Controls

We performed targeted PCR and Sanger sequencing of *LMP-1* in 414 eBL cases and 414 age- and geographically matched healthy controls enrolled in the EMBLEM study in Uganda, Tanzania, and Kenya during 2010-2016 (34). The cases and controls had comparable mean age (7.25 in cases versus 7.73 years in healthy controls). PCR was done using Lei-1 primer pair (Lei-F: GCCTCCGGCAGACCCCGCAAATC; Lei-R: GGGCAAAGGGTGTAATACTTAC), which targets a 435 base pair amplicon of the *LMP-1* promoter and exon 1 hypervariable region (43). Approximately 100-300 ng of genomic(g) DNA was used as an input template (25). PCR mixtures were prepared using 10 µL 2× DreamTaq Master Mix (Thermo Scientific, USA), 0.5 µL primers (10 μM each), and gDNA template. Nuclease-free water was added to the mixture to attain a final test volume to 20 µl. Thermocycle was carried out in Eppendorf Mastercycler Pro S (Eppendorf North America, Hauppauge, NY, USA) using initial denature at 94°C for 5 min, thermocycle at 94°C for 30 s, 55°C or 60°C for 30 s, and 72°C for 30 s for a total of 45 cycles, followed by a final extension at 72°C for 7 min. The PCR products were separated by electrophoresis using pre-made 2% agarose gel pre-stained with ethidium bromide in 1× Tris-Acetate EDTA (TAE) buffer. The amplicons were visualized under blue light at wavelength 460–520 µm (Amersham Imager 600, GE Healthcare, Marlborough, MA, USA). The result of the sample was classified as EBV PCR positive or negative. EBV PCR negative samples were not tested further. The amplicons matching the desired length were retrieved from the EBV PCR positive samples by eluting from the agarose gel using QIAquick Gel Extraction kit (Qiagen, San Jose, CA, USA) and stored in nuclease-free water. The retrieved amplicons were subjected to bi-directional Sanger sequencing reactions implemented by Macrogen Inc. (Macrogen, Rockville, MD, USA). The chromatograms generated from sequencing were exported into CLC Genomics Workbench Version 20.0.4 and BioEdit v7.0 with the Clustal W algorithm to visualize, assemble, and align the sequence file against the EBV WT reference genome (26).

### Ethics Approval and Consent to Participate

The data from BLGSP were accessed with permission from dbGap (Approval #50629-8 and #52320-7 for project #12922) to investigate genomic variation of EBV in the BL. The EMBLEM study was performed with approval from ethics committees at Uganda Virus Research Institute (GC/127), Uganda National Council for Science and Technology (H816), Tanzania National Institute for Medical Research (NIMR/HQ/R.8c/Vol. IX/1023), Moi University/Moi Teaching and Referral Hospital (000536), and National Cancer Institute (10-C-N133). Written informed consent was obtained from the guardians of the participants and assent from participants aged 7 years or older.

### Statistical Methods

We used phylogenetic trees to explore and describe EBV genomic variation. The association of EBV positivity and successful sequencing of *LMP-1* and identified patterns with eBL case status was calculated using frequency tables and logistic regression to calculate odds ratios and 95% confidence intervals (ORs, 95% CIs). EBV infection in Africa occurs during infancy (44, 45) and is lifelong (46). Thus, the reference category for the pattern analysis comprised EBV PCR positive patients regardless of sequencing result. PCR-positive but sequence-negative patients were considered infected, but the infection was low titer, presumably because it was virologically controlled and below sequencing sensitivity and probably irrelevant for BL risk (44, 45). The associations were adjusted for sex, age group, *falciparum* infection status, anemia [as an indicator of malaria burden (47)], and area of residence.

## Results

### Characteristics of the Compiled EBV Genome Datasets and Phylogenetic Patterns

**Table 1** summarizes the characteristics of the 730 EBV genomic sequences compiled in the present study while the details of the sequences, including accession numbers, EBV type, and geographical origin, are shown in **Supplementary Table S1**. About half of the compiled sequences were from BL [n=176, 24.1%, including 77 newly added EBV genomic sequences extracted from the BLGSP **Supplementary Table S2** (7)] or NPC (n=162, 22.2%). Most (161 of 176) BL samples were from Africa; only 15 BL samples were from from outside Africa, including two samples from Asia. Although NPC occurs in Africa, none of the NPC samples studied were from Africa. EBV type 1 accounted for 82.9% of the compiled samples, EBV type 2 accounted for 8.6% of the samples and type was recorded as unknown in 8.5% of the samples. From the full set, we selected 219 EBV sequences (**Table 1**, sample details in **Supplementary Table S3**) for whole genome-wide phylogenetic analysis (see *Methods*, above), of which 130 (59.3%) were from BL or from Africa. All the EBV genomic sequences from Africa included in this set were from BL patients (either primary biopsies, BL-derived cell lines, or normal samples from peripheral blood or buccal). We filtered samples from healthy people because they lacked sufficient EBV read depth. EBV genomic samples from non-African origin were sampled manually by clade to provide context for the comparative analysis.

**Table 1:** Characteristics of whole EBV genomes analyzed for phylogenetic pattern and EBV patterns.

Characteristic	Selected genomes N=219	Total genome set N=730
**EBV type**		
** Type 1**	191 (87.2%)	605 (82.9%)
** Type 2**	21 (9.6%)	63 (8.6%)
** Unknown**	7 (3.2%)	62 (8.5%)
**New genomes**		
** BLGSP**	74 (33.8%)	77 (10.5%)
**Previously published**	145 (66.2%)	654 (89.5%)
**Phenotype**		
**B95-8**	3 (1.4%)	4 (0.5%)
**Endemic Burkitt lymphoma**	128 (58.4%)	176 (24.1%)
**Sporadic Burkitt lymphoma**	2 (0.9%)	2 (0.3%)
**EBV + lymphoid malignancy**	1 (0.5%)	14 (1.9%)
**Gastric cancer**	19 (8.7%)	26 (3.6%)
**Hodgkin lymphoma**	7 (3.2%)	15 (2.0%)
**Nasopharyngeal carcinoma**	15 (6.8%)	162 (22.2%)
**Healthy donor**	10 (4.6%)	82 (11.2%)
**Lymphoblastoid cell lines (LCLs)**		26 (3.6%)
**NK/T cell lymphoma**	5 (2.3%)	25 (3.4%)
**PTLD**	4 (1.8%)	33 (4.5%)
**Chronic active EBV infection**	14 (6.4%)	115 (15.7%)
**Infectious mononucleosis**	6 (2.7%)	15 (2.0%)
**Extra nodal NK/T cell lymphoma/leukemia**	4 (1.8%)	26 (3.6%)
**Lympho-epithelioma**	1 (0.5%)	2 (0.3%)
**Unknown**		25 (3.4%)
**EBV *LMP-1* Pattern**		
**A**	46 (21.0%)	85 (11.6%)
**AB**	20 (9.1%)	26 (3.6%)
**B**	79 (36.1%)	398 (54.5%)
**C**	0 (0%)	7 (0.9%)
**D**	36 (16.4%)	98 (13.4%)
**F**	23 (10.5%)	28 (3.8%)
**G**	0 (0%)	8 (1.1%)
**H**	5 (2.3%)	12 (1.6%)
**I**	5 (2.3%)	5 (0.7%)
**J**	1 (0.5%)	6 (0.8%)
**Unclassified (but cluster with Pattern B)**	4 (1.8%)	57 (7.8%)

Of 730 EBV genomic samples identified, 431 were high-quality with genome size >170 kbp and gap <2000 ambiguous nucleotides. Because we were specifically interested in exploring genomic patterns of samples from Africa and facing the limitation of computational power for aligning whole genome sequences of a large dataset, we selected 219 EBV samples, including all qualifying samples from BL or Africa plus around 35% sequences selected from all other qualifying samples (see **Figure 1**).

**Figure 3:**
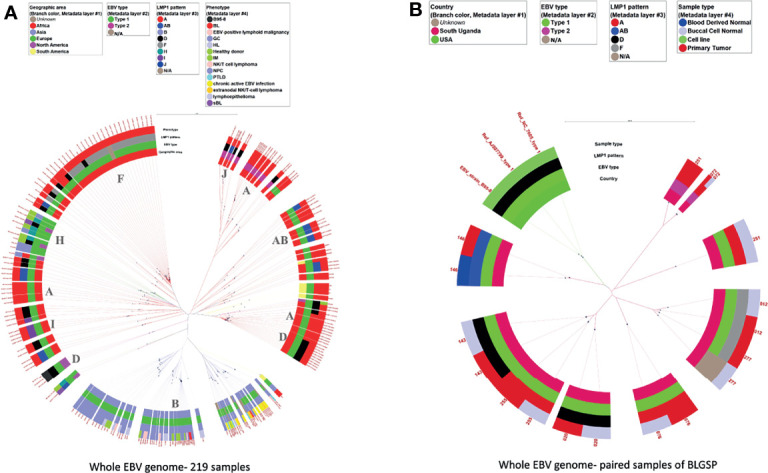
Phylogenetic tree of whole EBV genomes from samples with various conditions and from different geographic areas. **(A)** 219 whole EBV genomes, **(B)** 9 tumor-normal pairs of the BLGSP dataset. The sample conditions were color-coded. The rings from the inner side to the outer side are annotations for the Geographic area, EBV type, *LMP-1* pattern, and phenotype of each sample. The missing data were tan color. The black dots indicate the positions of each sample away from the root (center). The scale bar value for distancing: **(A)** 0.006 **(B)** 0.009. The dominated *LMP-1* pattern of the corresponding clade was annotated in the inner circle. The color of the extension line of each sample is consistent with the color of the Geographic area. Three genomic sequences of EBV type 1 obtained from GenBank, including the original NC_07605 derived from B95-8 cell line and genomic sequencing datasets of the same cell line by 2 other different labs, were used as references for analytic classification in Figure **(B)**.

**Figure 3A** shows the phylogenetic tree for the 219 genomic sequences with the layers of the circle (inside to outside) showing the geographic origin, EBV type, *LMP-1* pattern, and phenotype of the sample from which the EBV sequence was obtained. The scale bar of phylogenetic distance (0.006) indicates high similarity of the sequences of EBV genomes. The tree shows, as has been reported in several previous reports (24, 38, 40, 41, 48), that the sequences of EBV from Africa are genetically separate from those in Asia. The phylogenetic tree shows four major genetic branches in EBV from Africa and two genetic branches in EBV from Asia, of which one branch splits into two sub-branches. When *LMP-1* patterns were considered (see details below), the African EBV samples carried eight *LMP-1* patterns. These patterns were found, mostly, but not always, on different tree branches. EBV type 1 samples from Africa showed imperfect clusters that corresponded to AB, H, and I, while Patterns A and D were carried by samples belonging to different tree branches. EBV type 2 samples formed two sub-clusters, which carried Pattern A and Pattern J *LMP-1* variants, respectively. Interestingly, three EBV type 1 samples carrying Pattern A variants clustered close to the EBV type 2 samples that also carried Pattern A *LMP-1* variants. Of the EBV type 1 samples, Asian samples formed two separate tree branches, of which one branch split into two sub-branches. These Asian samples all carried Pattern B *LMP-1* variants, regardless of the tree branch. Most EBV from South America intermixed with those from Africa and carried Pattern A *LMP-1* variants, while those from Europe and North America also intermixed with samples from Africa, but appeared to carry a distinct *LMP-1* H pattern. (**Figure 3A**). However, a smaller set of EBV from South America, Europe, or North America intermixed with samples from Asia, carrying either Pattern A, AB, or H *LMP-1* variants (**Figure 3A**). These cluster patterns suggest that there are distinct genetic subgroups of EBV in the samples from Africa and Asia consistent with the idea of the presence of EBV phylogroups by Zanella et al. (41). The two sequences from BL samples in Asia intermixed with Asian samples, but on different tree branches of the Asian sequences, and they all carried Pattern B *LMP-1* variants suggesting that Pattern B is a prominent geographic marker of EBV from Asia (**Figure 3A** and **Supplementary Table S3**).

**Figure 3B** shows the phylogenetic tree of EBV from nine paired tumor-normal samples from BLGSP patients who had sufficient EBV genome coverage in WGS for phylogenetic analysis in both samples to detect possible co-infection by multiple EBV strains. The EBV WGS sequence was identical in tumor and buccal cells in eight patients, but discordant in one patient (**#251**) who had type 1 EBV in the tumor and type 2 EBV in the buccal sample. This patient’s EBV viral load in tumor and buccal samples were high with more than 1700x and 3800x genome coverage-depth of the EBV genome sequence reads in WGS of the BL tumor and buccal samples, respectively (**Figure 3B** and **Table 5**).

**Figure 4:**
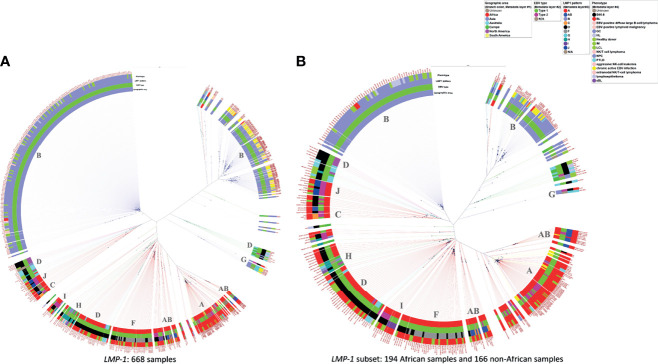
Phylogenetic tree of *LMP-1* sequences from samples with various conditions and from different geographic areas. **(A)** 668 available sequences of *LMP-1*, **(B)** 360 *LMP-1* sequences from 194 African samples and 166 non-African samples for lowering the graphic density for better visualization. The rings from the inner side to the outer side are annotations for the Geographic area, EBV type, *LMP-1* pattern, and phenotype of each sample. The black dots indicate the positions of each sample away from the center. The scale bar value for distancing: **(A)** 0.022 **(B)** 0.035. The dominant *LMP-1* pattern of the corresponding clade was annotated in the inner circle. The color of the extension line of each sample is consistent with the color of the Geographic area.

The findings based on WGS genomic sequences noted above were similarly observed in phylogenetic trees using only the *LMP-1* genomic sequence including a large set of 668 sequences and a subset of 360 genomic sequences (**Figure 4A**, **Supplementary Tables S4** and **Figure 4B**, **Supplementary Tables S5**). The *LMP-1* results confirm WGS genomic sequence patterns that EBV from Asia clustered on two main branches, of which one branch forms at least two sub-branches. These Asian samples were mostly homogenous in their *LMP-1* pattern, which was Pattern B except for a small set of samples classified as Pattern G. Consistent with WGS results, the Asian EBV sequences from tumors appear to cluster separately from those from non-tumor samples (**Figure 4A** **),** suggesting that recent efforts to sample populations without malignancy in Asia are starting to pay dividends in terms of separating tumor versus non-tumor EBV in population data.

Our parallel phylogenetic analysis of *EBNA1* **(** **Supplementary Figures 1A, B** **)** and *EBNA2* (**Supplementary Figures 2A, B**) genes confirmed the general impression that EBV from Africa tumors/populations is separate from EBV in Asia and that the *LMP-1* patterns identified are independent of sequence variations at those loci, and, by extension, EBV type.

### *LMP-1* Variants and Patterns in Representative EBV Samples From GenBank

We identified 281 SNVs (details in **Supplementary Tables S10** and **S11**) in the *LMP-1* hypervariable region of 597 sequences curated when compared to the WT B95-8 reference genome. These included 83 (30%) SNVs that formed 12 *LMP-1* patterns (A to L, as classified in **Supplementary Table S12** with representative examples in **Supplementary Table S13**). This study expands the number of *LMP-1* patterns from four reported in the original publication by Lei et al. (25) to 12. The original *LMP-1* patterns (A-D) in Lei et al. were formed by 55 SNVs (25). In this study, we identified 28 new SNVs that form eight novel patterns that are consistently found in many samples. One of the new patterns is a hybrid of A and B SNVs. Although this pattern may have resulted from recombination, we did not identify a hard transition from A to AB because the pattern-forming SNVs are scattered over a long stretch of the *LMP-1* sequence. The split between A and B SNVs was such that about 50% of pattern A SNVs were retained at the 5’ end and 50% of pattern A SNV’s at the 3’ end were replaced by pattern B SNVs. Representative pattern AB samples are shown in **Table S13** with a blue-gray shade (and also in **Supplementary Table S10**) for additional guidance. This hybrid pattern was observed in samples from Africa but not in those from Asia.

We also observed five new patterns (E, F, G, H, I, and J) based on being observed consistently in many samples (**Supplementary Tables S10** and **S12**). Patterns K (A-70G and position C-9T) and L (A+28T in the promoter region) are provisional because they are based on EMBLEM samples that were tested using only Lei-1 PCR primers. These primers target only variants in *LMP-1* exon and therefore generate a sequence that is insufficient to categorize SNVs in the *LMP-1* core promoter region (2 SNVs, see **Table S12**) to the *LMP-2B* exon 1 (6 SNVs, see **Table S12**) to exclude alternative pattern I and pattern J. We also note that pattern E is defined by one non-synonymous variation in amino acid position I63L (ATA>CTA), which was observed in many EMBLEM samples and considered valid. Pattern G was characterized by 4 variations at the promoter region and 3 non-synonymous variations found in exons 1, 2, and 3. Pattern H was characterized by variation at amino acid position G82A (GGC > GCC). It is possible that some patterns with SNVs in relatively adjacent positions (e.g., D, E, H, I or J and K) might belong to single clusters, which will become clearer as more samples are studied.

Two *LMP-1* patterns (B, and G) were observed principally in samples from Asia, whereas the other ten patterns were observed principally in non-Asian origin samples. Each pattern exhibited sub-patterns that will require further research to identify those that represent lineages versus artifacts (**Supplementary Tables S11** and **13** **)**. We also noted many variations, some of which were common and others rare, but not contributing to a pattern or sub-pattern.

Among BL, 92% of samples belong to one of four *LMP-1* patterns; about half were either A or AB (33.3% and 15.7%, respectively) and the remainder were D and H (24.5% and 18.4%, respectively). We observed the four *LMP-1* patterns to predominate in BL samples with WGS genomic sequences, i.e., convenient samples (**Table 2**) or in the nine samples from BLGSP patients, which are well-characterized from two different regions in Uganda (**Table 3**) (7). Among the BLGSP patients with paired tumor-normal (buccal or blood) samples, EBV loads were evidently higher in the buccal than in peripheral blood cells of these patients (**Table 3**). EBV sequence reads could be found with more than 100-fold genome coverage in 6 out of 9 buccal specimens from the BL patients in the BLGSP with paired tumor-buccal samples, but in none of the 14 peripheral blood samples from the BLGSP patients (**Table 3**). Only 2 blood samples had more than 5-fold EBV genome coverage in approximately 80 WGS of the blood-related samples (**Supplementary Table S2**, the average coverage of the 2 blood samples were highlighted with orange).

**Table 2:** *LMP-1* promoter and coding pattern variations in the EBV genomes in 114 primary tumor or cell lines with WGS data.

Characteristic		
*LMP-1* variant pattern	Number	Percentage
**A**	38	33.3%
**B**	2	1.8%
**AB**	18	15.7%
**D**	28	24.6%
**F**	21	18.4%
**I**	4	3.5%
**J**	1	0.8%
**Not Applicable**	2	1.8%
**Total**	114	100%

Details of samples included in this analysis are in **Supplementary Table S3**.

**Table 3:** EBV *LMP-1* patterns in 23 subjects with whole genome sequence extracted from BLGSP, including 14 with samples in EMBLEM who were also genotyped in this study using the Sanger method.

Case ID	WGS depth		EBV type	*LMP-1* Pattern from WGS		*LMP-1* pattern from Sanger
	Tumor	Normal		Tumor	Normal	Normal
Nine Paired BLGSP samples of tumor and buccal (normal) samples*						
**BLGSP-71-06-00012**	1525.09	10.20	1	F	F	–
**BLGSP-71-06-00020**	549.83	569.85	1	D	D	–
**BLGSP-71-06-00072**	773.10	724.81	2	AI	AI	–
**BLGSP-71-06-00076**	1129.61	144.91	1	AIII	AIII	–
**BLGSP-71-06-00143**	1275.68	240.18	1	D	D	–
**BLGSP-71-06-00146**	655.55	27.22	1	ABIII	ABIII	–
**BLGSP-71-06-00251**	1708.80	3848.28	1/2	AIII	AVI	–
**BLGSP-71-06-00255**	2972.55	358.95	1	AIII	AIII	–
**BLGSP-71-06-00277**	3965.62	15.03	1	F	n/a	–
**14 paired BLGSP samples of tumor and blood (normal) samples and Sanger data from *LMP-1* gene targeted PCR sequencing in the EMBLEM study**						
**BLGSP-71-08-00033**	1492.4	1.1	2	D	n/a	D
**BLGSP-71-08-00036**	645.5	1.0	2	D	n/a	D
**BLGSP-71-08-00038**	1055.9	1.6	1	D	n/a	AB7
**BLGSP-71-08-00041**	1766.1	1.3	1	D	n/a	A3
**BLGSP-71-08-00050#**	1424.7	4.3	n/a	ABII	n/a	ABII
**BLGSP-71-08-00191#**	3.6	1.8	n/a	NA	n/a	n/a
**BLGSP-71-08-00194**	2705.2	15.2	1	AIII	AIII	n/a
**BLGSP-71-08-00197**	3053.0	32.6	1	I	I	K
**BLGSP-71-08-00199**	2914.7	3.6	1	I	n/a	n/a
**BLGSP-71-08-00200**	1526.9	2.9	1	D	n/a	D
**BLGSP-71-08-00204**	2818.9	2.5	1	ABII	n/a	AB7
**BLGSP-71-08-00205**	1116.6	3.4	1	D	n/a	n/a
**BLGSP-71-08-00206**	3558.9	2.9	1	I	n/a	I
**BLGSP-71-08-00210**	1776.2	3.1	1	D	n/a	K

*The last three digits of the samples used to annotate the phylogenetic tree in **Figure 3** **B**.

#These samples could not be classified for EBV type due to insufficient sequence coverage in EBNA-2.

n/a, Not applicable because sequence data was insufficient to genotype samples for EBV LMP-1 patterns.

We had paired tumor-peripheral blood results for 14 patients included in the BLGSP and the EMBLEM studies. Two subjects who had sufficient depth of EBV WGS genome coverage (>5-fold genome depth) had concordant EBV *LMP-1* patterns between tumor samples in the BLGSP and blood samples in the EMBLEM. Of those, 12 patients with insufficient EBV genome coverage in WGS of their blood samples in the EMBLEM, EBV *LMP-1* patterns from Sanger sequencing were concordant with tumor in six patients, discordant in three, and not determined in blood in three patients (**Table 3**). These results are difficult to interpret because of low Sanger sequencing quality in blood samples with apparent very low EBV titers.

### Associations of eBL With Peripheral Blood EBV and *LMP-1* Patterns

**Table 4** shows the characteristics of the eBL cases and age- and geographically matched healthy controls in EMBLEM who were studied using PCR and Sanger sequencing. **Table 5** shows the associations with EBV positivity, successful sequencing of *LMP-1*, and with the identified patterns. EBV positivity was associated with being an eBL case (95.6% of eBL cases versus 79.2% of controls, aOR =3.83; 95% CI 2.06-7.14). Among the EBV positives, successful Sanger sequencing was associated with being an eBL case (66.7% of eBL cases versus 29.6% of healthy controls, aOR= 8.27; 95% CI 5.27-13.0) (**Table 5**). Among EBV positives, detection of four *LMP-1* patterns (A, AB, D, and K) was associated with being an eBL case (63.1% of the eBL cases versus in 27.1% of controls. The association was significant for each of the patterns (aOR= 11.4, 95% CI 5.89-22.0 for A; aOR= 5.58, 95% CI 2.62-11.9 for pattern AB; aOR=7.67, 95% CI 4.09-14.4 for pattern D; and aOR=7.90, 95% CI 3.98-15.7 for pattern K) using the subjects who were EBV positive as the reference group.

**Table 4:** Demographic and clinical characteristics of the participants in the EMBLEM Study population.

Characteristic	Cases, n (%)	Controls, n (%)	P value*
**All subjects**	414 (50.0%)	414 (50.0%)	
**Gender**			0.050
**Females**	168 (40.6%)	196 (47.3%)	
**Males**	246 (59.4%)	218 (52.7%)	
**Age, years (mean +/- SD)**	7.24 (3.66)	7.73 (3.33)	0.043
**Age group, years**			0.073
**0-2**	38 (9.2%)	23 (5.6%)	
**3-5**	107 (25.8%)	90 (21.7%)	
**6-8**	125 (30.2%)	131 (31.6%)	
**9-11**	77 (18.6%)	101 (24.4%)	
**13-16**	67 (16.2%)	69 (16.7%)	
**Country**			0.52
**Uganda**	213 (51.5%)	214 (51.7%)	
**Tanzania**	89 (21.5%)	100 (24.1%)	
**Kenya**	112 (27.0%)	100 (24.1)	

*Computation of percentages includes categories with missing information, but computation of p-values excludes those subjects.

**Table 5:** Associations of *LMP-1* viremia with endemic Burkitt lymphoma in the EMBLEM Study.

Characteristic	eBL cases (%)	Controls (%)	Crude OR (95% CI)	Adjusted OR (95% CI)*
**EBV PCR**				
**Negative**	18 (4.4%)	86 (20.8%)	Ref.	Ref.
**Positive**	396 (95.6%)	328 (79.2%)	5.76 (3.40-9.79)	3.83 (2.06-7.14)
**EBV Sequence**				
**Not successful**	132 (33.3%)	231 (70.4%)	Ref.	Ref.
**Successful**	264 (67.7%)	97 (29.6%)	4.76 (3.47-6.53)	8.28 (5.27-13.0)
**EBV Pattern**				
**Sequencing not successful**	132 (33.3%)	231 (70.4%)	Ref.	Ref.
**A**	73 (18.4%)	18 (5.5%)	7.09 (4.06-12.4)	11.4 (5.89-22.0)
**A/B**	43 (10.9%)	19 (5.8%)	3.96 (2.21-7.07)	5.58 (2.62-11.9)
**D**	82 (20.7%)	28 (8.5%)	5.12 (3.17-8.27)	7.68 (4.09-14.4)
**K**	52 (13.1%)	24 (7.3%)	3.79 (2.23-6.43)	7.90 (3.98-15.7)
**Other (E, I, L)**	14 (3.55%)	8 (2.4%)	3.06 (1.25-7.47)	7.98 (2.43-26.3)

*Adjusted for sex, age group (5 categories), P. falciparum infection, country, village characteristics (rural and proximity to surface water), and anemia (hemoglobin <11.6 g/dl).

## Discussion

We present a detailed phylogenetic analysis of EBV genomic sequences of samples obtained from Africa (primarily from BL patients) analyzed together with non-African EBV clades from elsewhere (26). Our EBV genomic and *LMP-1* findings confirm impressions from earlier studies (23, 24, 38, 40, 41, 48, 49) that EBV from Africa is genetically separate from EBV in Asia. Our results also confirm that there is extensive genetic diversity in *LMP-1*, as previously suspected (25–27). The results also suggest that only a fraction of the identified diversity in *LMP-1* is necessary to group into the 12 patterns i.e., based on ~30% of the 83 SNVs identified). The *LMP-1* patterns also showed consistent separation of Africa-versus Asia-origin samples. Our gene-specific analysis confirmed that the *LMP-1* patterns were unique and not phylogenetically related to *EBNA-1* or *EBNA-2*, or EBV type. *LMP-1* analysis identified 9 patterns distributed across four WGS phylogenetic tree branches in the African-type samples. By comparison, we identified two *LMP-1* patterns scattered across two WGS phylogenetic tree branches in the Asian EBV samples. The clear geographic patterns of *LMP-1* patterns are interesting given the geographic eBL and NPC risk profiles in Africa versus China (33). *LMP-1* patterns may have potential utility as biomarkers to study geographical variants of EBV at relatively low cost using *LMP-1* PCR and Sanger sequencing in epidemiological studies such as EMBLEM (34, 50), where large-scale use of WGS is not feasible.

We identified dual infection in WGS data (EBV type 1 and type 2) and *LMP-1* patterns using PCR and Sanger (D and A or AB). These findings are likely valid because they were observed in high viral titers samples and they are consistent with earlier reports that have reported dual infection in some individuals (51). The observation of multiple type or variant infections adds complexity to the interpretation of EBV genomic variation in epidemiological and clinical studies when assays yield conflicting results in patients. The results also raise a concern about which body compartments (buccal or blood), should be targeted in epidemiological studies of disease patterns to identify valid or the strongest associations. Our finding that PCR-sequencing was more successful in buccal than peripheral blood samples (**Table 3**) suggests that buccal samples may be preferable for the study of non-malignant samples, although further research is needed to clarify performance issues.

Our results suggest that 92% of BL patients carry one of four *LMP-1* patterns (A, AB, D, and F), and 50% of them carry either A or AB. Because these patterns are rare or not observed at all in Asia, they fit the hypothesis that these EBV variants may be both geographic and tumor markers. However, these results while conclusive about the geographic association, they are inconclusive about phenotypic association because the EBV data from Africa are mostly from BL patients with little representation of healthy populations from Africa. For example, only 40 of 668 *LMP-1* sequences analyzed in this study were from healthy people in Africa versus 130 from BL patients. BL develops in about 0.005% of EBV-infected people, suggesting that current EBV data are not representative of EBV in the general population without BL (12) and the geographic and phenotypic associations with *LMP-1* are confounded. The finding that most BL carried one of four *LMP-1* patterns, which are virtually absent in Asia (25, 26), suggests that investigating the distribution of these markers in healthy people in Africa is a promising area of research.

Our finding that different EBV variants are found in different geographic regions is similar to the pattern reported for other viral carcinogens, such as the human papillomavirus (HPV) (52, 53) and hepatitis B virus (HBV) (54). Multiple carcinogenic variants are known for HPV, and different types are found in different geographic areas (HPV genotypes 35 and 45 predominate in Africa, whereas HPV genotypes 52 and 58 predominate in Asia) (55). Similarly, multiple genotypes exist for HBV with genotypes A and E predominating in Africa, whereas genotypes B and C predominate in Asia (54). These geographical patterns are important for public health, biology, and diagnosis. They reflect underlying immunological pressure that drives diversification through host-pathogen adaptations in populations living in geographically separate areas, with some of the best characterized examples being HIV (56), HCV (57), multiple bacterial pathogens (58), and plasmodia (59). Because *LMP-1* is a target of the host immune response (60, 61), it is possible that *LMP-1* diversification reported here is driven by immunological pressure and has led to diversification of *LMP-1* patterns. We noted that some *LMP-1* patterns were found in different branches of the WGS phylogenetic tree, including Pattern A and D found both in type 1 and type 2 EBV, while others were not found on other branches or types. We speculate that this intermixture of *LMP-1* patterns in different tree branches may be because *LMP-1* patterns represent an early gene sequence that preceded the modern sequences observed in EBV type 1 or 2. These patterns could have been evolutionarily preserved due to essential biological function favoring their preservation.

EBV is a suitable target for discovery biomarkers for diagnosis (62) and study of the etiology of BL (25, 26). The *LMP-1* region has been an attractive locus to characterize EBV’s biological, genetic, and epidemiological properties (63, 64). *LMP-1* has been linked to biological changes that influence transmission, transformation, and tumor microenvironment (65). Phylogenetic studies have revealed distinct phylogroups of EBV (23, 24, 38, 40, 41, 48, 49), and principle components analysis of WGS data has identified SNVs that are correlated with ancestry (48). However, access to HTS is still limited, especially for large epidemiological studies conducted in Africa, where eBL is a public health problem. This compelled us to investigate whether the *LMP-1* patterns described by Lei et al. might be sufficient characterize geographic or phenotypic patterns of EBV. We developed Lei-1, Lei-2, and Lei-3 primers for a simple and cost-effective PCR and Sanger sequencing assay (25, 26). Our results using only Lei-1 primers in EMBLEM confirms that assay can be used to type *LMP-1* patterns, but the results with one primer are insufficient to resolve some patterns that may have SNVs in regions not covered by Lei-1 primers. We also identified differential completion rates in eBL cases versus healthy controls as a limitation in samples with low EBV viral titers. EBV establishes lifelong and low-grade infection (1-50 infected B cells per million) that is maintained in most healthy individuals (66), so all our subjects were infected but those with low viral titer cannot be typed, making it difficult to distinguish between clearance, persistence, and poorly controlled infection.

Our study is subject to several limitations, despite its use of large current EBV genome datasets. First, EBV HTS data are skewed to cancer patients (tumor or normal samples) with gross under-representation of healthy people. This bias in EBV sampling was observed for BL and healthy people from Africa, but we also noted it to be significant samples from Europe, North America, and astonishingly extreme for certain regions of Asia, such as India. Because HTS datasets are likely to play an important role in the discovery and fine mapping of carcinogenic EBV variants, this issue requires urgent attention through collaboration between scientists with access to populations and those with access to HTS technology and computational resources. Second, the N-J methods used to infer phylogeny may be less accurate than other methods such as the Maximum Likelihood (ML) methods (24). We used the NJ methods because they are reasonable for initial quick exploration of data and hypothesis generation, and they yield robust results across a range of small to large datasets and suffer only a small decline in accuracy across that range (42). We acknowledge that our results are not complemented by mechanistic explanations about the functional implications of the *LMP-1* patterns on EBV biology, virus-host interaction, transmission, or cell transformation. The epidemiological scope of our studies precluded mechanistic studies, but we hope that the findings will inspire those studies.

The strengths of our study are that we used a larger set of samples from Africa to study *LMP-1* patterns as potential biomarkers of EBV genetic diversity. The results support further optimizing the *LMP-1* PCR-Sanger sequencing assay for use as a relatively low-cost assay to investigate the geographic and phenotypic associations with EBV-related disease. Further research is needed to improve the success rate of this assay in normal samples with low viral loads.

To conclude, the phylogenetic analysis of EBV focusing on samples from Africa or BL confirms that EBV from Africa is genetically separated from EBV in Asia. We show that *LMP-1* patterns cluster separately for African versus Asian samples, with European, North American, and South American samples clustering mostly, but not exclusively, with EBV from Africa. Four EBV *LMP-1* patterns accounted for most EBV genotypes in BL patients, but these results may still reflect geographic patterns of EBV because EBV samples from Africa were mostly from BL patients and with few samples from the general population. Our findings suggest *LMP-1* variants are promising markers for identifying and classifying EBV genetic variants in quantitative and qualitative research to identify EBV variants associated with EBV-related cancer, including eBL.

## Data Availability Statement

The datasets for this study can be found in the links provided in **Supplementary Table S1** and **Table S2**. The EMBLEM data and code used in the current analysis will be made available upon request from the corresponding author. The MSA for the 219 samples included in WGS phylogenetic analysis as well as the samples used for *LMP-1*, *EBNA-1*, and *EBNA-2* phylogenetic analysis can be accessed at the following link: https://github.com/smbulaiteye/EBVBL_Africa_focus.git.

## Ethics Statement

The EMBLEM study was performed with approval from ethics committees at Uganda Virus Research Institute (GC/127), Uganda National Council for Science and Technology (H816), Tanzania National Institute for Medical Research (NIMR/HQ/R.8c/Vol. IX/1023), Moi University/Moi Teaching and Referral Hospital (000536), and National Cancer Institute (10-C-N133). Written informed consent to participate in this study was provided by the participants’ legal guardian/next of kin.

## Author Contributions

S-CL and SM conceived and designed the experiments. H-ML, P-JC, G-CH, and BL performed the experiments and analyzed the data; ST contributed materials and analysis tools. IO, IL, MO, SR, PK, CT, PW, RK, WW, NM, EK, LA, RMP, KB, and JG performed fieldwork. H-ML drafted the manuscript. S-CL, SM, and JG critically edited the manuscript. All authors reviewed and approved the final manuscript.

## Funding

This project was supported in part by an appointment to the Research Fellowship Program at the Office of Tissue and Advanced Therapies/Center for Biologics Evaluation and Research, U.S. Food and Drug Administration (FDA), administered by the Oak Ridge Institute for Science and Education through an interagency agreement between the U.S. Department of Energy and FDA. The fieldwork was funded by the Intramural Research Program of the Division of Cancer Epidemiology and Genetics, National Cancer Institute (NCI) (Contracts HHSN261201100063C and HHSN261201100007I) and, in part, by the Intramural Research Program, National Institute of Allergy and Infectious Diseases (SR), National Institutes of Health, Department of Health and Human Services, and the laboratory work by an interagency agreement between the National Cancer Institute and FDA (PUR4763663).

## Conflict of Interest

The authors declare that the research was conducted in the absence of any commercial or financial relationships that could be construed as a potential conflict of interest.

## Publisher’s Note

All claims expressed in this article are solely those of the authors and do not necessarily represent those of their affiliated organizations, or those of the publisher, the editors and the reviewers. Any product that may be evaluated in this article, or claim that may be made by its manufacturer, is not guaranteed or endorsed by the publisher.

## Supplementary Material

Supplementary Figure 1 Phylogenetic tree of *EBNA-1* sequences from samples with various conditions and from different geographic areas. **(A)** 705 available sequences of *EBNA-1*, **(B)** 378 *EBNA-1* sequences from 210 African samples and 168non-African samples for lowering the graphic density for better visualization. The rings from the inner side to the outer side are annotations for the Geographic area, EBV type, *LMP-1* pattern, and phenotype of each sample. The black dots indicate the positions of each sample away from the center. The scale bar value for distancing: **(A)** 0.013 **(B)** 0.030. The dominant *LMP-1* pattern of the corresponding clade was annotated in the inner circle. The color of the extension line of each sample is consistent with the color of the Geographic area.

Supplementary Figure 2 Phylogenetic tree of *EBNA-2* sequences from samples with various conditions and from different geographic areas. **(A)** 595 available sequences of *LMP-1*, **(B)** 299 *EBNA-2* sequences from 143 African samples and 156 non-African samples for lowering the graphic density for better visualization. The rings from the inner side to the outer side are annotations for the Geographic area, EBV type, *LMP-1* pattern, and phenotype of each sample. The black dots indicate the positions of each sample away from the center. The scale bar value for distancing: **(A)** 0.022 **(B)** 0.010. The dominant *LMP-1* pattern of the corresponding clade was annotated in the inner circle. The color of the extension line of each sample is consistent with the color of the Geographic area.
