# Supplementary figures and images for: Epstein-Barr Virus in Burkitt Lymphoma in Africa Reveals a Limited Set of Whole Genome and LMP-1 Sequence Patterns: Analysis of Archival Datasets and Field Samples From Uganda, Tanzania, and Kenya
Source: Front Oncol. 2022 Mar 7;12:812224. doi: 10.3389/fonc.2022.812224 (PMC8948429; doi:10.3389/fonc.2022.812224)

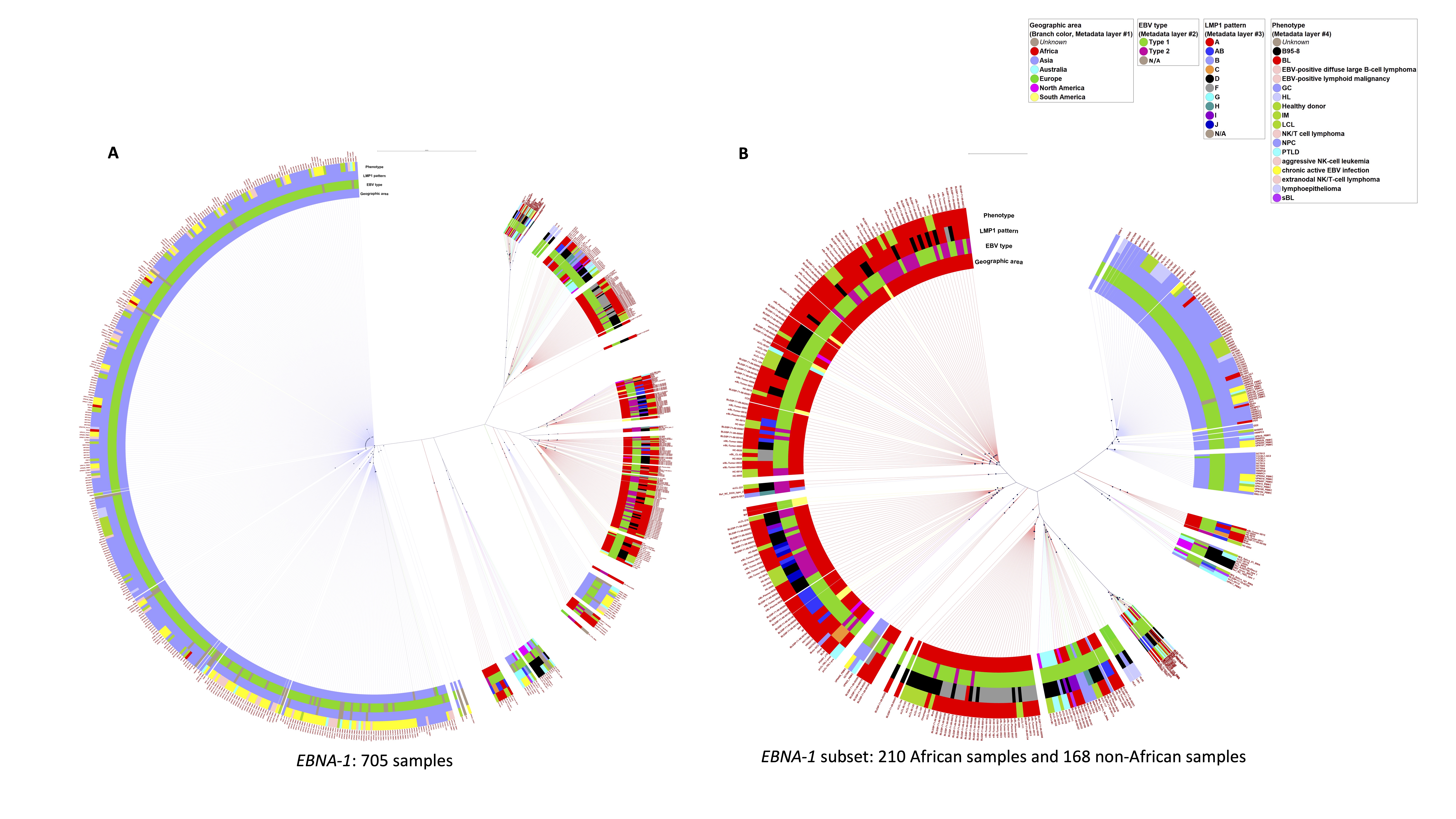

Supplement: Supplementary Figure 1 — Phylogenetic tree of EBNA-1 sequences from samples with various conditions and from different geographic areas. (A) 705 available sequences of EBNA-1, (B) 378 EBNA-1 sequences from 210 African samples and 168non-African samples for lowering the graphic density for better visualization. The rings from the inner side to the outer side are annotations for the Geographic area, EBV type, LMP-1 pattern, and phenotype of each sample. The black dots indicate the positions of each sample away from the center. The scale bar value for distancing: (A) 0.013 (B) 0.030. The dominant LMP-1 pattern of the corresponding clade was annotated in the inner circle. The color of the extension line of each sample is consistent with the color of the Geographic area. [file Image_1.jpeg]

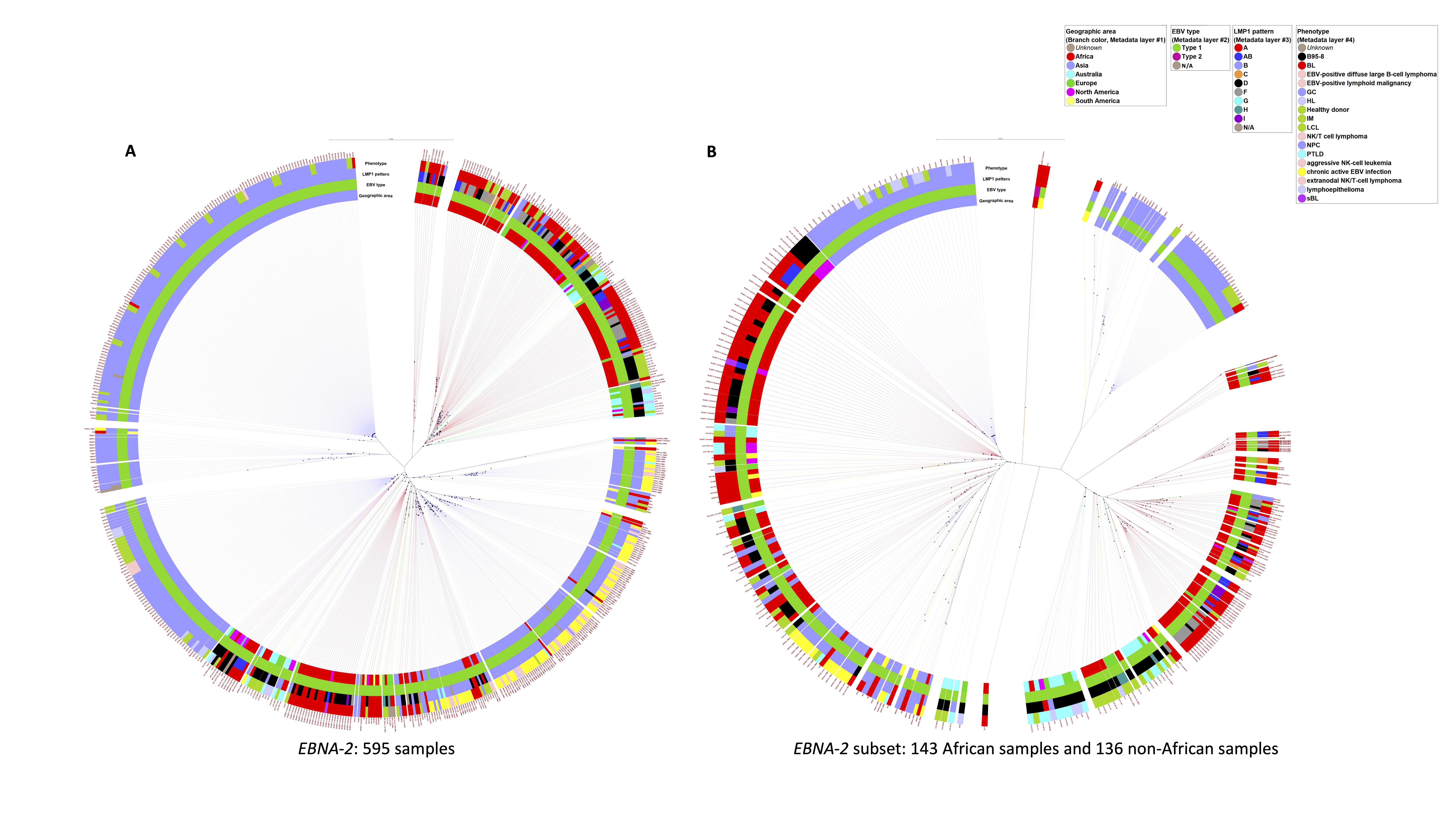

Supplement: Supplementary Figure 2 — Phylogenetic tree of EBNA-2 sequences from samples with various conditions and from different geographic areas. (A) 595 available sequences of LMP-1, (B) 299 EBNA-2 sequences from 143 African samples and 156 non-African samples for lowering the graphic density for better visualization. The rings from the inner side to the outer side are annotations for the Geographic area, EBV type, LMP-1 pattern, and phenotype of each sample. The black dots indicate the positions of each sample away from the center. The scale bar value for distancing: (A) 0.022 (B) 0.010. The dominant LMP-1 pattern of the corresponding clade was annotated in the inner circle. The color of the extension line of each sample is consistent with the color of the Geographic area. [file Image_2.jpeg]
